# Validation study of the safety attitudes questionnaire (SAQ) in public hospitals of Heilongjiang province, China

**DOI:** 10.1371/journal.pone.0179486

**Published:** 2017-06-21

**Authors:** Ying Li, Xiaowen Zhao, Xue Zhang, Chi Zhang, Hongkun Ma, Mingli Jiao, Xia Li, Lijun Gao, Mo Hao, Jun Lv, Yanming Zhao, Yu Cui, Jinghua Liu, Zhaoquan Huang, Wuxiang Shi, Qunhong Wu, Mei Yin

**Affiliations:** 1 Department of Health Policy and Hospital Management, School of Public Health, Harbin Medical University, Harbin, Heilongjiang province, China; 2 Department of Health Economics, School of Public Health, Harbin Medical University, Harbin, Heilongjiang province, China; 3 School of Humanities and Social Sciences, Harbin Medical University, Harbin, Heilongjiang province, China; 4 People's Hospital of Taizhou City, Taizhou, Jiangsu province, China; 5 Harbin Medical University, Harbin, Heilongjiang province, China; 6 Institute of Quantitative and Technical Economics, Chinese Academy of Social Science, Beijing, China; 7 College of Pharmacy, Harbin Medical University, Harbin, Heilongjiang province, China; 8 Department of Social Medicine, School of Public Health, Harbin Medical University, Harbin, Heilongjiang province, China; 9 School of Public Health, Fudan University, Shanghai, China; 10 Department of CT, The 2nd Affiliated Hospital of Harbin Medical University, Harbin, Heilongjiang province, China; 11 Department of general medicine, School of Public Health, Qiqihar Medical University, Qiqihar, Heilongjiang province, China; 12 School of Health Management, Guilin Medical University, Guilin, Guangxi, China; Universita degli Studi di Firenze, ITALY

## Abstract

**Purpose:**

The objective of this study was to validate the reliability and validity of the safety attitudes questionnaire (SAQ) in Heilongjiang province, northern China.

**Methods:**

The SAQ was distributed to 27 public hospitals in five cities across Heilongjiang province. The Cronbach’s α, item–dimension and dimension–dimension correlations were calculated. Descriptive analyses and confirmatory factor analysis were also performed.

**Results:**

The recovery rate of the questionnaire was 84.45%. The validity and reliability measures of the SAQ were acceptable. The goodness-of-fit index from the confirmatory factor analysis showed a reasonable model fit (CFI = 0.93, GFI = 0.91, RMSEA = 0.05). The Cronbach’s α value for the scale was 0.91 and ranged from 0.66 to 0.91 for each of the scales. The SAQ showed good internal consistency reliability.

**Conclusion:**

The SAQ had satisfactory psychometric properties and could be a useful tool to measure safety attitudes in public hospitals in Heilongjiang province in China.

## Introduction

Despite the continuous improvement of medical technology, many people are still treated with the risk of harm. Patient safety is an important aspect of healthcare organisations. Studies in some countries have shown that a lack of attention to patient safety will lead to medical errors, which may result in further injury to patients and increase hospital stays [1, 2]. Patient safety is an important component of healthcare quality[3], and this has been highlighted by healthcare organisations following the Institute of Medicine’s (IOM) report, ‘To Err is Human: Building a Safer Health System’[4]. This report emphasised that adverse events can be reported without blaming individuals and when mistakes occur, lessons ought to be learned. Thus, an overarching culture of patient safety has been suggested as a core organisational mechanism of hospitals to promote safe, effective, and timely healthcare[5]. Indeed, patient safety culture can have a critical impact on the safety of healthcare environments[6]. Patient safety culture was defined by the British Health & Safety Commission as ‘the product of individual and group values, attitudes, perceptions, competencies, and patterns of behaviour that determine the commitment to, and the style and proficiency of an organisation’s safety management’[7].

In the recent years, greater attention has been paid to the development of patient safety cultures, given that patient safety issues are becoming more valued by countries throughout the world. Adequate assessment is a prerequisite of research on patient safety culture. To this end, instruments have been developed to measure patient safety culture[8–11], but only the Safety Attitudes Questionnaire (SAQ) has been linked to patient outcomes[12]. Furthermore, the psychometric properties of the SAQ have been established in many countries including the United States (English version)[12], Switzerland (German version)[13], Taiwan (Chinese version)[14], and Norway (Norwegian version)[15]. It has not been validated in northern China, however.

The geographic positions and cultures of different regions of China and other countries vary considerably. Thus, this study targeted public hospitals in Heilongjiang province, northern China. The aim of this study was to translate the SAQ and test its reliability and validity in this region. The results of this study will provide opportunities for follow-up studies.

## Materials and methods

### Safety attitudes questionnaire

The predecessor of the SAQ was used to measure airline safety cultures and was called the Flight Management Attitude Questionnaire. Subsequently, the questionnaire was introduced to the medical industry as a tool for evaluating the safety culture of medical institutions by Sexton et al.[15, 16]. The original SAQ was developed by Sexton in 2000, which comprises 60 items, 30 of which are the core items[12]. After continuous study of the SAQ, a universal version, called the SAQ Short Form, was created; this version comprises a total of 6 dimensions and 30 items. Based on this survey, the selected instrument for data collection was the short version of the generic SAQ, which included 30 items [12]. The SAQ includes six dimensions, including teamwork climate, safety climate, job satisfaction, perception of management, stress recognition, and working conditions, each of which consists several items. Additional questions were added to assess respondents’ demographic information (e.g. gender, age, profession). A study conducted in Switzerland introduced the SAQ and expanded the 5-point Likert scale by adding a ‘not applicable’ option[13]. This study adopted the modification. So all items were rated on a 5-point Likert scale (1 = disagree strongly, 2 = disagree slightly, 3 = neutral, 4 = agree slightly, 5 = agree strongly) and a ‘not applicable option’ was included for each item. The SAQ was chosen as the evaluation tool because it is one of the most commonly used and rigorously validated tools for measuring the safety climate in healthcare[17]. To make the SAQ more consistent with the original questionnaire in terms of concepts, connotation, and culture, the original English-language version was translated, back-translated, and reviewed by a panel of experts[18]. Four experts with backgrounds in medical and public health management translated the original questionnaire into Chinese. Then, the items were translated by English speaking medical graduates into English to confirm that the meaning of the original questionnaire was unchanged. Five experts consisting of a public health professional, nurse, physician, researcher, and healthcare administrator then reviewed the questionnaire and offered feedback to ensure cross-cultural consistency and applicability in China. With the first draft of the questionnaire, a survey among Harbin medical staff, who had worked for more than five years, was conducted. Based on the analysis of the survey results, inappropriate parts of the questionnaire were revised to arrive at the final version.

### Sample

A cross-sectional survey was conducted in July and August 2014 by contacting 2,000 healthcare workers. The total population of Heilongjiang in 2014 was 38.33 million. According to ‘Statistical yearbook of health and family planning in China (2014)’, there are 301 secondary hospitals and 82 tertiary hospitals in Heilongjiang province. According to the geographical distribution, time, and resources, we investigated the areas in the north (Jiamusi), south (Mudanjiang), west (Qiqihar and Daqing), and centre (Harbin) of the province. Purposive sampling was used to select 35 hospitals. However, only 27 hospitals (15 tertiary hospitals and 12 secondary hospitals) agreed to participate in the study, including general hospitals, specialised hospitals, and traditional medicine hospitals, all of which were public hospitals.

All hospitals provided permission to conduct the investigation. With the assistance of hospital managers, the questionnaire was distributed during staff meetings at the hospital outpatient departments, inpatient departments, medical technology departments, and some administrative departments. All respondents were hospital workers and able to influence and be influenced by the hospital’s patient safety culture. Respondents were informed of the general content and purpose of the survey, and they voluntarily completed the questionnaire. Participants were encouraged to ask for clarification if they did not understand the questionnaire. Hospital managers were asked to request the healthcare workers to complete any outstanding questionnaires. Completed questionnaires were collected seven days after they had been distributed. Questionnaires were considered valid if at least 70% of the items were completed. Questionnaires were collected and stored by the management team. Individuals who could not complete the questionnaire on time were asked to mail the questionnaire to the research team in order to improve the response rate.

### Data analysis

All data were analysed using SAS 9.3 (SAS Institute, Cary, NC, USA). Descriptive statistics (means and standard deviations for quantitative data, and frequencies and percentages for qualitative data) were used to describe the demographic characteristics, items, and factors. Mean scores and standard deviations (SD) were calculated for all SAQ items. Scores greater than 3 were considered positive responses. The rate of positive responses was calculated to obtain an indication of positive patient safety attitudes in the sample. The positive response rate was also used to evaluate attitudes toward patient safety culture on different dimensions and items. The scores on negatively worded items (e.g. ‘In this clinical area, it is difficult to speak up if I perceive a problem with patient care’, ‘In this clinical area, it is difficult to discuss errors’) were reversed before the analysis.

Pearson’s correlation coefficients were calculated to describe the relationships of different factors as well as items with corresponding factors. Cronbach’s α coefficient was used to evaluate the reliability of the SAQ, and confirmatory factor analysis (CFA) was used to evaluate the validity. Statistical significance was defined as *P* < 0.05.

The laboratory protocols of this study have been deposited in protocols.io (DOI: http://dx.doi.org/10.17504/protocols.io.h5cb82w).

### Ethical issues

This study was based on data regarding patient safety culture among healthcare workers. Responding to the questionnaire was anonymous. All participants were informed about the purpose of the study in advance, and data were collected voluntarily. The study was approved by the Institutional Review Board of Harbin Medical University.

## Results

### Demographics

A total of 2,000 questionnaires were distributed to 27 hospitals and 1,689 valid responses were returned (response rate 84.45%). The average age of the respondents was 34.76. Among the respondents, 463 (27%) were males and 1,219 (72%) were females, 703 (42%) were physicians, 712 (42%) were nurses, and 109 (6%) were technicians. Most of the healthcare staff had worked for their current hospital for five years or more. Table 1 shows the detailed demographic characteristics.

**Table 1:** Demographic characteristics of respondents (N = 1,689).

Characteristic		Mean (SD)	Frequency (n)	Percentage
**Age (years)**		34.76±9.08		
**Gender**	Male		463	0.27
	Female		1219	0.72
	Missing		7	0.01
**Profession**	Physician		703	0.42
	Nurse		712	0.42
	Technician		109	0.06
	Administrator		102	0.06
	Other		35	0.02
	Missing		28	0.02
**Years in current hospital**	≤1		185	0.11
	1–5		484	0.29
	6–10		323	0.19
	11–15		163	0.1
	16–20		213	0.12
	≥21		305	0.18
	Missing		16	0.01

### Descriptive statistics

Item responses on the SAQ are shown in Table 2 (items 2 and 11 are reverse-scored). The means for the 30 items ranged from 3.02 to 4.27, with SD between 0.75 and 1.17. Item 6 (‘The physicians and nurses here work together as a well-coordinated team’) had the highest mean (4.27), and item 11 (‘In this clinical area, it is difficult to discuss errors’) had the lowest (3.02). The number of missing responses and items answered with *not applicable* are also shown in Table 2 and ranged from 0.2% to 12.5%. Item 2 (‘In this clinical area, it is difficult to speak up if I perceive a problem with patient care’), from the teamwork climate dimension, had the highest proportion of not applicable answers (12.5%), while item 18 (‘Morale in this clinical area is high’), from the job satisfaction dimension, had the lowest proportion (0.2%). The number of positive responses (items answered with *agree slightly* and *agree strongly*) ranged from 564 (33%) to 1,402 (83%), thus most items were positively responded. Correlation coefficients for the relationship between each item and the corresponding dimensions ranged from 0.19 to 0.92 (*P* < 0.01). Item 2 (‘In this clinical area, it is difficult to speak up if I perceive a problem with patient care’) and item 11 (‘In this clinical area, it is difficult to discuss errors’) had item–dimension correlations of below 0.30, indicating a weak relationship with the other items. The correlations of the other items and dimensions were better (0.68–0.92). The item factor loadings were between 0.09 and 0.91. Both item 2 and item 11 had factor loadings below the acceptable threshold of 0.3. Detailed information is shown in Table 2.

**Table 2:** SAQ items descriptions (N = 1,689).

Dimension, item number, and item text	Missing data and answered *not applicable* (%)	Mean (SD)	NPR	NPR (%)	Item-dimension correlation	Item factor loading
**Teamwork climate**						
1. Nurse input is well received in this clinical area.	31 (0.018)	3.81 (0.93)	1,070	0.63	0.73	0.69
2. In this clinical area, it is difficult to speak up if I perceive a problem with patient care. (R)*	211 (0.125)	3.41 (1.16)	845	0.5	0.27	0.09
3. Disagreements in this clinical area are appropriately resolved.	30 (0.018)	4.04 (0.87)	1,242	0.74	0.73	0.69
4. I have the support I need from other personnel to care for patients.	21 (0.012)	3.91 (0.89)	1,150	0.68	0.72	0.71
5. It is easy for personnel in this clinical area to ask questions when there is something that they do not understand.	50 (0.03)	4.03 (0.84)	1,232	0.73	0.75	0.73
6. The physicians and nurses here work together as a well-coordinated team.	32 (0.019)	4.27 (0.75)	1,402	0.83	0.68	0.63
**Safety climate**						
7. I would feel safe being treated here as a patient.	18 (0.011)	3.83 (0.9)	1,085	0.64	0.73	0.64
8. Medical errors are handled appropriately in this clinical area.	15 (0.009)	3.9 (0.83)	1,178	0.7	0.8	0.82
9. I know the proper channels to direct questions regarding patient safety in this clinical area.	23 (0.014)	3.76 (0.89)	1,012	0.6	0.79	0.82
10. I receive appropriate feedback about my performance.	17 (0.01)	3.68 (0.97)	981	0.58	0.73	0.72
11. In this clinical area, it is difficult to discuss errors. (R)*	84 (0.05)	3.02 (1.13)	564	0.33	0.19	0.12
12. I am encouraged by my colleagues to report any patient safety concerns I may have.	17 (0.01)	3.73 (0.9)	998	0.59	0.69	0.63
13. The culture in this clinical area makes it easy to learn from the errors of other.	11 (0.007)	3.9 (0.86)	1,185	0.7	0.75	0.73
**Job satisfaction**						
14. I like my job.	13 (0.008)	3.79 (1)	1,063	0.63	0.81	0.75
15. Working in this hospital is like being part of a large family.	6 (0.004)	3.91 (0.9)	1,166	0.69	0.87	0.85
16. This is a good place to work.	5 (0.003)	3.73 (0.99)	1,011	0.6	0.84	0.81
17. I am proud to work in this clinical area.	5 (0.003)	3.82 (0.95)	1,068	0.63	0.92	0.91
18. Morale in this clinical area is high.	3 (0.002)	3.74 (0.98)	1,009	0.6	0.86	0.81
**Stress recognition**						
19. When my workload becomes excessive, my performance is impaired.	50 (0.03)	3.46 (1.13)	851	0.5	0.85	0.77
20. I am less effective at work when fatigued.	18 (0.011)	3.71 (1.07)	1,054	0.62	0.87	0.84
21. I am more likely to make errors in tense or hostile situations.	38 (0.023)	3.47 (1.17)	883	0.52	0.91	0.87
22. Fatigue impairs my performance during emergency situations.	30 (0.018)	3.59 (1.13)	965	0.57	0.91	0.91
**Perception of management**						
23. Management supports my daily efforts.	16 (0.01)	3.72 (0.9)	944	0.56	0.85	0.77
24. Management does not knowingly compromise the safety of patients.	19 (0.011)	3.9 (0.81)	1,183	0.7	0.82	0.74
25. I get adequate, timely information about events in the hospital that might affect my work from the unit management.	17 (0.01)	3.75 (0.87)	1,015	0.6	0.88	0.83
26. The levels of staffing in this clinical area are sufficient to handle the number of patients.	38 (0.023)	3.64 (0.93)	928	0.55	0.82	0.73
**Working conditions**						
27. This hospital does a good job of training new personnel.	21 (0.012)	3.99 (0.83)	1,237	0.73	0.81	0.72
28. All the necessary information for diagnostic and therapeutic decisions is routinely available to me.	9 (0.005)	4.07 (0.87)	1,285	0.76	0.87	0.82
29. Trainees in my discipline are adequately supervised.	7 (0.004)	4 (0.91)	1,240	0.73	0.87	0.83
30. Problem personnel in this clinical area are dealt with constructively by our management.	17 (0.01)	4.04 (0.89)	1,255	0.74	0.84	0.78

*Note*: *SD*, standard deviation; *NPR*, number of positive responses (including *agree slightly* and *agree strongly*)

(R)*, reverse-scored item.

### Internal consistency

The Cronbach’s α was used to measure the internal consistency of the scale items. For the whole scale, Cronbach’s α was 0.91 and ranged between 0.66 and 0.91 for the six dimensions, indicating the strong reliability of the SAQ. The Cronbach’s α coefficients for job satisfaction and stress recognition were both 0.91. Coefficients for all dimensions and total scales are shown in Table 3.

**Table 3:** SAQ dimension descriptions and scale reliability (N = 1689).

Dimensions	Cronbach’s α
1. Teamwork climate	0.66
2. Safety climate	0.76
3. Job satisfaction	0.91
4. Stress recognition	0.91
5. Perceptions of management	0.86
6. Working conditions	0.87
Whole scale	0.91

Pearson’s correlation coefficients were used to describe the correlations between the different dimensions. This analysis indicated that all scales were positively related, and the correlation coefficients ranged from 0.09 to 0.58 (*P* < 0.01). However, the stress recognition dimension was less relevant to other dimensions. The correlation coefficients are shown in Table 4.

**Table 4:** Inter-correlations of SAQ dimensions (N = 1,689).

Dimensions	1	2	3	4	5	6
1. Teamwork climate	1					
2. Safety climate	0.54	1				
3. Job satisfaction	0.47	0.58	1			
4. Stress recognition	0.11	0.19	0.1	1		
5. Perception of management	0.39	0.54	0.51	0.09	1	
6. Work conditions	0.54	0.57	0.49	0.14	0.45	1

*Note*: All coefficients were significant at the *P*< 0.0001 level.

### Factor structure of the SAQ

The confirmatory factor analysis (CFA) showed that each of the six dimensions (teamwork climate, safety climate, job satisfaction, stress recognition, perceptions of management, and working conditions) fitted the data well and indicated a good model fit for the overall safety construct. For the six dimensions, CFI = 0.93, GFI = 0.91, and RMSEA = 0.05. The goodness-of-fit indices for these six dimensions are presented in Table 5, and *P*-values were significant for all factors (*P* < 0.05).

**Table 5:** Results of confirmatory factor analysis (CFA) (N = 1,689).

Dimensions	Model-fit indices			
	CFI	GFI	RMSEA	NFI
1. Teamwork climate	0.94	0.96	0.11	0.93
2. Safety climate	0.96	0.97	0.08	0.96
3. Job satisfaction	0.99	0.99	0.07	0.99
4. Stress recognition	0.97	0.96	0.19	0.97
5. Perceptions of management	0.98	0.98	0.15	0.98
6. Working conditions	0.93	0.99	0.08	0.99
Whole scale	0.93	0.91	0.05	0.91

*Note*: *CFI*, comparative fit index; *GFI*, goodness-of-fit index; *RMSEA*, root mean square error of approximation; *NFI*, normal fit index.

## Discussion

This cross-sectional study assessed the psychometric properties of the SAQ in Heilongjiang province using a purposively selected sample. The response rate of 84.45% was the highest among similar studies, in which rates were 59% –78.6% [5–7, 13, 15, 17, 19, 20]. The rate was also better than that found in a study in Taiwan (69.4%)[14]. Because studies on patient safety culture in Heilongjiang are not very common, and patients’ safety in the hospital is also considered as a sensitive topic in today's society. Medical staffs of these hospitals showed interest in this study and were willing to cooperate with us. This study is also their usual demand. In addition, the cooperation of the hospital managers during the course of our investigation and the rigour of the process led to an increase in the rate of positive responses. The highest percentage of the missing and *not applicable* answers for an item (‘It is easy for personnel in this clinical area to ask questions when there is something that they do not understand’) was 12.5%, which was less than the acceptability rate (13%)[19]. Therefore, no items were removed in the analyses, and previous studies have shown similar results[19, 21–23].

### Internal consistency

In this study, the Cronbach’s α coefficient was used to test the reliability of the scale. The Cronbach’s α for the total scale was high (0.91), and removing any items from the analysis only caused minor improvements to this score. Values close to 1 indicate a high internal consistency of the scale. Cronbach’s α coefficients higher than 0.8 indicate excellent internal consistency, 0.6–0.8 are good, and lesser than 0.6 are poor[24]. The values of other studies ranged from 0.56 to 0.89, suggesting acceptable levels of internal consistency [12–15, 17, 19, 21–23]. Thus, this study had better results than the previous studies. Furthermore, the relatively high Cronbach’s α values demonstrated a good internal consistency on all scale dimensions, that is, all values were between 0.76 and 0.91, except for teamwork climate (0.66), which was still within the acceptable range. The result of lower Cronbach’s α value for teamwork climate is similar to previous studies on Norwegian and Swiss samples [13, 15]. One possible reason for this relatively poor, albeit still acceptable, reliability may be that the second item on this dimension was inversely scored. It may have been difficult for the respondents to understand the reverse rating of this item. They often queried about the meaning of this negative item. Therefore, it is likely that it may have been interpreted incorrectly by some respondents.

The factor loadings of item 2 (‘In this clinical area, it is difficult to speak up if I perceive a problem with patient care’) and item 11 (‘In this clinical area, it is difficult to discuss errors’) were 0.09 and 0.12, respectively. These values were below the acceptable threshold of 0.03[19], which indicated that they had a weak relationship with the other items on the scale. Notably, the factor loading for item 2 was similar to that obtained in studies in Denmark and Turkey [19, 25]. It would thus be necessary to study these two items further in a future study. The correlations of each item with the total score of its dimension (*P* < 0.01) were significant. The correlation coefficients of the two negative items with their respective dimensions were small. A possible reason is that the medical staff did not understand the reverse-scored items well enough. This result is similar to that of the samples from Sweden and Denmark[19, 21]. However, one study of Swedish pharmacies showed that measurement results did not differ regardless of positive or negative wording[21]. Further research of this item is required, including item translation, back-translation, and item-content descriptions. For the dimension–dimension correlations, strong positive correlations were found for the most part, although stress recognition had a somewhat weaker correlation with the other dimensions. Similar results have been found in a previous study[14, 17]. Therefore, further validation is needed for this dimension.

### Factor structure of the SAQ

The content validity of the whole questionnaire was confirmed as good by experts, indicating that the items accurately expressed the intended content. The construct validity, by contrast, refers to whether the questionnaire as a whole measures the theoretical concept it is designed to measure. This can be judged by the goodness-of-fit indicators in a confirmatory factor analysis. The six-dimensional model (teamwork climate, safety climate, job satisfaction, stress recognition, working conditions, and perceptions of management) fitted the data well. Furthermore, the CFI (0.93) was better than what it was in other studies and exceeded the threshold value[26]. The CFI of other studies ranged from 0.9 to 0.99[12–15, 25, 27], while the RMSEA ranged from 0.03 to 0.07[12–15, 25, 27]. In this study, the RMSEA was 0.05, which is better than the values found in a Danish study (0.053)[19] and a Taiwanese study (0.06)[14]. One of the possible reasons for this is that different countries use a slightly different SAQ.

### Limitations of the study

Due to time and resource restrictions, this study has the limitation of using purposive sampling for recruiting individuals from Heilongjiang province alone rather than using systemic sampling. The findings of this study may not be generalised to all hospitals in Heilongjiang province. It is necessary to explore more effective research methods for safety attitudes questionnaire. The cluster effect of each healthcare setting may exist, which may affect the results of reliability and validity. Therefore, this problem should be considered in the selection of hospitals and the process of data analysis.

## Conclusions

Based on this investigation of medical staff in public hospitals in Heilongjiang province, a version of the SAQ with acceptable psychometric properties was obtained, including good reliability and acceptable validity. However, due to the differences in geographic positions and cultures among different regions in China and other countries, including Taiwan (China), further research and discussion should be conducted to confirm whether the stress recognition dimension should be deleted. Furthermore, the reverse-scored items need to be studied in the future due to the low positive response rates and factor loadings. This study is an initial step to establishing the psychometric properties of the SAQ, and further research is needed to refine the instrument and re-examine its psychometric properties in the light of these results. The study of patient safety culture will be an important motivator for policymakers and managers to improve patient safety. This study is the first step in the patient safety culture research. However, this step is very important. Validating the SAQ has laid a solid foundation for research on the status quo of patient safety culture, and future studies could explore the strengths and weaknesses of patient safety culture in hospitals, and draw attention of hospital managers, health employees, people related to patient safety culture education, and other stakeholders to patient safety culture.

## Supporting information

S1 File The dataset and descriptions underlying the findings in our study was supplied in the supplemental files. (XLSX)

## References

[1] De Vries EN, Ramrattan MA, Smorenburg SM, Gouma DJ, Boermeester MA. The incidence and nature of in-hospital adverse events: a systematic review. Quality & Safety in Health Care. 2008;17(3):216–223. 18519629 10.1136/qshc.2007.023622 PMC2569153

[2] Huang DT, Clermont G, Kong L, Weissfeld LA, Sexton JB, Rowan KM, et al Intensive care unit safety culture and outcomes: a US multicenter study. International Journal for Quality in Health Care. 2010;22(3):151–161. doi: 10.1093/intqhc/mzq017 20382662 10.1093/intqhc/mzq017 PMC2868527

[3] Cooper JB, Gaba DM, Liang B, Woods D, Blum LN. The National Patient Safety Foundation agenda for research and development in patient safety. Medgenmed Medscape General Medicine. 2000;2(3):E38 11104484

[4] Kohn LT, Corrigan JM, Donaldson MS. Institute of Medicine). To err is human: building a safer health system. Annales Francaises D Anesthesie Et De Reanimation. 2000;7(Dec):245–246.

[5] Ostroff C, Kinicki AJ, Tamkins MM. Organizational Culture and Climate 2003 565–593.

[6] Essawi M. Organizational Culture and Leadership. 2013.

[7] Horlock JH, Broadbent DE. Acsni Study Group on Human Factors: 3rd Report. 1993.

[8] Singer SJ, Gaba DM, Geppert JJ, Sinaiko AD, Howard SK, Park KC. The culture of safety: results of an organization-wide survey in 15 California hospitals. Quality & Safety in Health Care. 2003;12(2):112–118. 12679507 10.1136/qhc.12.2.112 PMC1743680

[9] Weingart SN, Farbstein K, Davis RB, Phillips RS. Using a multihospital survey to examine the safety culture. Joint Commission Journal on Quality & Safety. 2004;30(3):125–132. 15032069 10.1016/s1549-3741(04)30014-6

[10] Sorra JS, Nieva VF. Reliability and validity of the Hospital Survey on Patient Safety Culture at a Norwegian hospital. 2004.

[11] Colla JB, Bracken AC, Kinney LM, Weeks WB. Measuring Patient Safety Climate: A Review of Surveys. Quality & Safety in Health Care. 2005;14(5):364–366. 16195571 10.1136/qshc.2005.014217 PMC1744072

[12] Sexton JB, Helmreich RL, Neilands TB, Rowan K, Vella K, Boyden J, et al The Safety Attitudes Questionnaire: psychometric properties, benchmarking data, and emerging research. BMC Health Services Research. 2006;6(1):: 44. 16584553 10.1186/1472-6963-6-44 PMC1481614

[13] Zimmermann N, Küng K, Sereika SM, Engberg S, Sexton B, Schwendimann R. Assessing the safety attitudes questionnaire (SAQ), German language version in Swiss university hospitals—a validation study. BMC Health Services Research. 2013;13(1):1–11. 24016183 10.1186/1472-6963-13-347 PMC3846625

[14] Lee WC, Wung HY, Liao HH, Lo CM, Chang FL, Wang PC, et al Hospital Safety Culture in Taiwan: A Nationwide Survey Using Chinese Version Safety Attitude Questionnaire. BMC Health Services Research. 2010;10(1): 234. 20698965 10.1186/1472-6963-10-234 PMC2924859

[15] Deilkås ET, Hofoss D. Psychometric properties of the Norwegian version of the Safety Attitudes Questionnaire (SAQ), Generic version (Short Form 2006). BMC Health Services Research. 2008;33(1):1820–1827. 10.1186/1472-6963-8-191 PMC2572612 18808693

[16] Euteneier MMA, Chop I, Eberlein-Gonska PMM. Analyse- und Reportingwerkzeuge: Springer Berlin Heidelberg; 2015.

[17] Gabrani A, Hoxha A, Simaku A, Gabrani J. Application of the Safety Attitudes Questionnaire (SAQ) in Albanian hospitals: a cross-sectional study. Bmj Open. 2015;5(4):775–780. 10.1136/bmjopen-2014-006528 PMC4401850 25877270

[18] Sperber AD. Translation and validation of study instruments for cross-cultural research. Gastroenterology. 2004;126(1 Suppl 1):124–128. 10.1053/j.gastro.2003.10.016 14978648

[19] Kristensen S, Sabroe S, Bartels P, Mainz J, Christensen KB. Adaption and validation of the Safety Attitudes Questionnaire for the Danish hospital setting. Clinical Epidemiology. 2015;7(default):49–58. 10.2147/CLEP.S75560 PMC4321416 25674015

[20] Guldenmund FW. (Mis)understanding Safety Culture and Its Relationship to Safety Management. Risk Analysis. 2010;30(10):1466–1480. doi: 10.1111/j.1539-6924.2010.01452.x 20626685 10.1111/j.1539-6924.2010.01452.x

[21] Nordénhägg A, Sexton JB, Kälvemarksporrong S, Ring L, Kettislindblad A. Assessing safety culture in pharmacies: the psychometric validation of the Safety Attitudes Questionnaire (SAQ) in a national sample of community pharmacies in Sweden. BMC Clinical Pharmacology. 2010;10(1): 8. 20380741 10.1186/1472-6904-10-8 PMC2868807

[22] Kho ME. Safety Climate Survey: reliability of results from a multicenter ICU survey. Quality & Safety in Health Care. 2005;14(4):273–278. 16076792 10.1136/qshc.2005.014316 PMC1744055

[23] Raftopoulos V, Savva N, Papadopoulou M. Safety Culture in the Maternity Units: a census survey using the Safety Attitudes Questionnaire. BMC Health Services Research. 2011;11(1):1161. 10.1186/1472-6963-11-238 PMC3196700 21951720

[24] Dov Z. Safety climate and beyond: A multi-level multi-climate framework. Safety Science. 2008;46(3):376–387.

[25] Kaya S, Barsbay S, Karabulut E. The Turkish version of the safety attitudes questionnaire: psychometric properties and baseline data. Quality & Safety in Health Care. 2010;19(6):572–577. 20671082 10.1136/qshc.2008.032003

[26] Bentler PM. Comparative fit indexes in structural models. Psychological Bulletin. 1990;107(2):238–246. 2320703 10.1037/0033-2909.107.2.238

[27] Göras C, Fan YW, Nilsson U, Ehrenberg A. Swedish translation and psychometric testing of the safety attitudes questionnaire (operating room version). BMC Health Services Research. 2013;13(1):1–7. 23506044 10.1186/1472-6963-13-104 PMC3618303

